# Cool

**Published:** 1844-08-10

**Authors:** 


### COOL.

*The Botanic Physicians of Rochester, N. Y.*, held a meeting on the 6th inst., at which, resolutions were passed in reference to the recent legislative enactments respecting medical practice. They represent the Legislature as having been actuated altogether in the matter by a conviction "of the importance and value of the reformed medical practice," and they accordingly pass resolutions of thanks for this special mark of favour. This is well enough, and what might have been expected; but the resolutions which follow, *renouncing* all connection professionally with mineral practitioners, as they call them, and declining under any circumstances to *consult* with them, although likewise characteristic, are absurd and deceptive, and must have appeared so even to those who voted for them.—*Ibid.*

